# From Deutsche Zeitschrift to International Journal of Legal Medicine—100 years of legal medicine through the lens of journal articles: Part 2: *Deutsche Zeitschrift für die gesamte gerichtliche Medizin* from 1948 to 1969

**DOI:** 10.1007/s00414-022-02912-z

**Published:** 2022-11-14

**Authors:** Ingo Wirth, Tony Fracasso, Heidi Pfeiffer, Andreas Schmeling

**Affiliations:** 1 Berlin, Germany; 2 University Center of Legal Medicine (CURML), Geneva University Hospital, Geneva, Switzerland; 3 Institute of Legal Medicine, University Hospital Münster, Münster, Germany

**Keywords:** History of legal medicine, Official publications, Academic articles

## Abstract

The interruption of the publication of the *Deutsche Zeitschrift für die gesamte gerichtliche Medizin* due to the war ended with volume 39 for the years 1948/1949. Until volume 66/1969, the journal appeared unchanged under the historical title. The 912 publications contained in the 28 volumes of these two decades cover topics from the main fields of forensic medicine, but also from related and unrelated disciplines. The topic-specific analysis of the publications shows a shift of the research focus in the German institutes since the post-war period. This is most evident in the decline in the number of publications from the fields of scientific and technical criminalistics as well as forensic psychiatry and psychology. An opposite trend with a significant increase in scientific papers was observed in alcohology, forensic genetics and traffic medicine. While the evaluated publications on most topics contain new findings that are still valid today, the use of blood group characteristics for forensic purposes came to an end as a result of the introduction of DNA analysis.

## Introduction

After a 4-year interruption, the 1st/2nd issue of the 39th volume of the *Deutsche Zeitschrift für die gesamte gerichtliche Medizin* (which, for brevity, we hereafter refer to as the *Deutsche Zeitschrift*) was completed on 25 June 1948. With its publication, the almost 100-year-old tradition of this periodical was revived [1]. As before, the Springer-Verlag and from now on also the Munich publishing house of J. F. Bergmann were involved in the publication. Up to volume 66/1969, 28 volumes of the *Deutsche Zeitschrift* were published by these two publishing houses, which during that time continued to be the official journal of the *Deutsche Gesellschaft für gerichtliche und soziale Medizin* (German Association of Forensic and Social Medicine). The 4th (and last) issue of the 66th volume with the year of publication 1969 is dated 2 March 1970. After the renaming of the subject of ‘Forensic Medicine’ (Gerichtliche Medizin) to ‘Legal Medicine’ (Rechtsmedizin), the journal retained its historical title for volumes 65 and 66/1969, but the subtitle was already *Organ der Deutschen Gesellschaft für Rechtsmedizin* (Official Journal of the German Association of Legal Medicine). From volume 67/1970 onwards, the journal also carried the new name in its title.

The first post-war volume was co-edited by the psychiatrist Hans Walter Gruhle, professor in Bonn, the director of the Munich Institute for Forensic Medicine Wolfgang Laves and Georg(e) Strassmann [2, 3], who had emigrated to the USA in 1938. For the following volume 40/1950–1951, they were already joined by Berthold Müller, Director of the Heidelberg Institute, and Victor Müller-Heß, professor at the Free University of Berlin, and from volume 42/1953–1954 on, Otto Schmidt, professor at Göttingen, was also involved in the editorial work. After Gruhle’s death in 1958, the editorial board was extended from volume 49/1959–1960 on to include expert representatives from all four German-speaking countries. Since then, Leopold Breitenecker from Vienna, Otto Prokop from Berlin, Fritz Schwarz from Zurich and the psychiatrist Max Müller from Bern were members of the board. The deaths of Müller-Heß in 1960 and Schmidt in 1962 reduced the size of the board, but no new editors joined. However, there were changes in the composition of the advisory board, which consisted of renowned experts and grew from 19 members in 1952, the year of foundation, to 33 in 1969.

In the first post-war volumes, the content was limited to original papers. The publication of congress reports was resumed in volume 43/1954–1955 with the report on the 32nd meeting of the Association held in Bonn in 1953 (43/85).^1^ With one exception, the conference reports were published in the *Deutsche Zeitschrift* until the 47th conference held in Innsbruck in 1968. The only missing event is the 40th conference held in Vienna in 1961, which took place in the framework of the Fifth International Congress of Forensic and Social Medicine [4]. From volume 44/1955–1956 onwards, conference lectures were also printed again. The present analysis wants to illustrate the scientific development of forensic medicine and related areas as reflected in the *Deutsche Zeitschrift*, so that only original articles and conference lectures from the proceedings of the Association edited for printing were taken into account.

## Analysis of academic articles

### The scientific focus in the period from 1948 to 1969

In accordance with the title of the journal, the volumes analysed cover the entire field of forensic medicine, but in several cases go far beyond the narrow scope of the subject. In order to obtain a representative result, the content analysis had to include not only the core topics of forensic/legal medicine, but also the full range of issues from related forensic sciences. In addition, individual papers on historical and epistemological problems, including personalia, were incorporated in the evaluation. The structure on which the analysis is based, with an overview of the number of publications on the individual topics in the period under review, can be found in Table 1.

**Table 1.** Overview of the number of publications on the individual topics in the period from 1948 to 1969

Topic	Number of publications
History and development of the discipline	
History of the discipline	2
Development of the discipline	2
Personalia	25
Legal issues Expert activities	24 12
General legal medicine	
External post-mortem examination/autopsy	17
Thanatology	53
Vital reactions	18
Forensic traumatology and pathology	
Forensic traumatology	149
Forensic obstetrics	24
Abortion	12
Filicide	1
Medical malpractice	21
Diseases and sudden natural death	50
Forensic toxicology	
Clinical signs of poisoning	37
Pathological anatomical findings	28
Toxicological chemical analyses	61
Alcohology	105
Identification of unknown decedents	19
Forensic genetics	
Blood group science	55
Forensic trace analysis	49
Parentage testing	41
Scientific-technical criminalistics	11
Clinical legal medicine	5
Forensic psychiatry and psychology	24
Sexual medicine	13
Traffic medicine	38
Social medicine	5
Criminology	11
Total	912

### History and development of the discipline

There are 2 publications on the *history of the discipline* from different historical sub-disciplines. Legal history includes Otto Schmidt’s study on ‘Forensic Medicine in the First Written Rights of Germanic Tribes’ (42/121). Wilhelm Hallermann’s work on the historical development of the Kiel Institute of Forensic Medicine can be classified as part of university history (45/247).

The decisive contribution to the *development of the discipline* was the renaming to legal medicine, which was decided on the occasion of the annual conference held in Innsbruck on 4 October 1968. The then acting chairman of the Association, Wolfgang Schwerd, explained the decision as follows: ‘The change of the discipline’s name, which had been discussed for quite some time, was made for factual, but also for linguistic reasons. The name “Forensic Medicine”, which has a long tradition, has been criticised time and again because it may undoubtedly create wrong ideas about the meaning of the discipline. […] The new name “Legal Medicine” is intended to provide a uniform name for the discipline and, of course, also for the specialised institutes’ (65/61).

### Personalia

In its capacity as the official publication of the Association, the *Deutsche Zeitschrift* was the periodical in which outstanding representatives of the Association and their life’s work were lauded. During the period under review, 23 obituaries of distinguished scholars from Germany and abroad were published, as well as 2 commemorations of milestone birthdays. Of these, the commemorative article by Victor Müller-Heß for his predecessor, ‘Fritz Strassmann, on his one hundredth birthday’ (48/177), deserves special mention. Strassmann had been deprived of all functions at the end of 1935 because of his Jewish descent [5]. When he died in 1940, no obituary was published in the *Deutsche Zeitschrift*, although he had been one of the co-editors since its foundation.

### Legal issues, expert activities

The publications on *legal issues* focus on criminal law and criminal procedure. Two publications dealing with criminal law illustrate the particular difficulties of fighting crime under the conditions of the post-war period. They deal on the one hand with problems of juvenile criminal law (41/267) and on the other hand with the fight against drug addiction (41/345). In fact, the 2 papers on the relevance of narcoanalysis (41/64, 43/552) also contain psychological reflections, but this manipulative technique is significant in terms of procedural law as a prohibited interrogation method under Sect. 136a of the Code of Criminal Procedure. The only other area of law that is represented several times is medical law, with 5 articles. In the only work on marriage law (45/420), the concepts of mental disorder and mental illness (§§ 44/45 Marriage Act) are explained. The Marriage Act, enacted as Control Council Act No. 16 on 20 February 1946, is an example of the elimination of National Socialist injustice after the end of the Second World War.

Apart from a legally oriented publication on the instruction prior to the expert assessment (58/176), the other 6 papers on *expert activities* concern concrete expert opinions, mostly on the question of the long-term consequences of blunt force trauma on the brain. Insurance law implications of various handicaps or medical conditions are the topic of 5 publications.

### General legal medicine

One article on the autopsy system belongs to the topic *external and internal post-mortem examination* (43/217). In it, George S. Strassmann explains the special features of the coroner and medical examiner system in the Anglo-American countries in comparison to the forensic practice in Central Europe. One of the two papers on autopsy technique deals with the examination methods for recording the pathomorphological findings after a whiplash injury of the cervical spine (64/204). Seven publications deal with supplementary examinations performed in addition to an autopsy. Apart from histological and microbiological methods, a versatile impression procedure (45/485) and a post-mortem angiography (62/199) are presented. Some morphological and functional properties of tissues and organs useful for the assessment of pathological autopsy findings are the subject of 6 studies, 3 of which concern the mechanical resilience of human skin.

The 9 publications on *early post-mortem changes* belong into the field of thanatology. Among the 3 papers on supravital reactions, there is a fundamental study on the idiomuscular bulge with a classification of mechanical muscle excitability (46/761). Apart from one study on the histology of livor mortis (40/499), the other publications on the early postmortem interval concern rigor mortis. In 1948, Wolfgang Laves presented an overview of the state of research on the development of posthumous rigidity with a critical evaluation of the lactic acid and ATP theory (39/186). Furthermore, one study was published on the relationship between post-mortem metabolism and rigor mortis in the cardiac muscle (53/163). Specifically on *late post-mortem changes*, 5 papers were found, of which the experimental studies on the decomposition processes in putrefaction and decay have provided fundamental insights into the nature of these two decomposition phenomena (41/236, 49/206). With 34 publications, studies on *thanatochemistry* form the largest group. Primarily, these are papers on blood chemistry, among which in turn studies on the sugar content of corpse blood and its diagnostic significance are particularly frequent. Hans-Joachim Wagner reported on the ‘Influence of antibiotics and sulfonamides on post-mortem physico-chemical processes’ (51/572). The results of his animal experiments with tetracycline suggested that the administration of antibiotics both reduced the bacterial flora involved in the processes of putrefaction and inhibited proteolytic enzymes. This showed an influence on rigor mortis, the resolution of which is considerably delayed.

Several publications make an attempt to find reliable *methods of estimating the time of death*. Various methodological approaches have been tried on the eye alone. While the usefulness of corneal opacity (48/80) and phosphate content of the aqueous humour (52/231) remained questionable, pH measurement in the vitreous body (64/110) proved to be completely unsuitable. On the basis of the experimental results, the authors also regarded the temperature of cardiac blood in the early post-mortem period (44/555) and the post-mortem cerebrospinal fluid pressure (52/273) as meaningless for practical purposes.

Most of the papers on *vital reactions* concern general reactions, of which embolisms are the most frequently discussed. Only one paper on bleeding to death was found on the other general vital reactions (40/617). Steffen Berg interpreted the different levels of adrenaline metabolites and histamine in corpse blood for different causes of death as a ‘vital reaction in the biochemical field’ (54/136). Local vital reactions are described in studies on the detection of vital muscle necroses after cut injuries (64/102) and on the differentiation of vital and post-mortem wounds by determining the esterase pattern of the skin (66/161). Age determination of injuries is the subject of 3 publications, including one on the examination of cannula punctures with enzyme-histochemical methods (64/252).

### Forensic traumatology and pathology

The human organism can be affected by the impact of *blunt force* in many ways. The most frequently described consequences of violence are head injuries. In the publications on skin wounds, the focus is on the identification of the inflicting tool based on the morphology of the wound. Of these, 2 publications present typical findings on the face and scalp when using guns as impact tools (44/50, 44/742). There are 3 publications on the types of skull fractures. Of traumatogenic significance are the two papers on ring fractures of the skull base due to a traction mode (51/601) and an extension mechanism (52/13) that had been largely ignored until then. In an experimental study on various test objects, the validity of Puppe’s rule on the priority of skull fractures could be confirmed (58/94). Most publications deal with the consequences of blunt force trauma to the brain. A problem discussed several times is the expert assessment of the so-called pachymeningitis haemorrhagica interna. Serious traumatic brain injuries caused by boxing matches are described in 3 papers. Not only were subdural haematomas detected (44/763), but intracerebral injuries and cerebral venous thromboses were also found several times (63/70, 66/75). As a contribution to the pathogenesis of contrecoup foci, a working group led by the pathologist Heinz David presented electron-microscopic findings on the cerebral cortex (56/177). Deaths after blunt trauma to the neck and trunk have also been reported. After a minor blow to the neck, there was a haemorrhage in the paraganglion caroticum, which was discussed as the cause of death (51/190). In all but one of the blunt thoracic traumas, an injury to the aorta was fatal. An experimental study of the mechanics of bone fracture in the lower leg was undertaken by Karl Sellier (56/341).

Among the comparatively few publications on the effects of *sharp force*, descriptions of stab wounds dominate. New insights into wound morphology are offered by work on injuries caused by scissors (45/53) and by knives with shaped blades (54/273), which was further supplemented with various stabbing tools in an extensive experimental study (62/4). An unusually shaped hand saw was used to dismember the victim after a homicide (50/592) and left well identifiable tool marks. In a detailed study with simulation tests, serial injuries proved to be characteristic for ship propellers (53/97). The effects of sharp force also include bite injuries, which were reported in a case history under the research question ‘Death by dog bites or post-mortem mangling’ (44/204).

The difficulties in reliably recognising mechanical *asphyxiation* are due to the fact that the external and internal findings on the corpse are only indicative. Consequently, attempts were made in various ways to find additional criteria to substantiate the suspected diagnosis. For example, there are reports of animal experiments with evidence of ‘giant cell formation during asphyxia’ (54/200) and of changes in carbohydrate and gas metabolism (56/421) as well as in the coagulation status and the cellular components of the blood during mechanical asphyxia (64/49). The only paper on *manual strangulation* deals with the microscopic findings on strangulation marks to distinguish them from skin lesions from other causes (45/17). On *ligature strangulation*, there was also only one article which emphasised the possibility of ligature strangulation without changes to the neck skin (58/190). A review of *death by hanging* discusses controversial questions of pathomechanism and then summarises that death by hanging ‘often becomes a very complicated syndrome’ depending on the different hanging situations (54/407). Causative factors include carotid artery compression, functional anatomical features of the basal cerebral vessels, airway constriction, pressure of the noose on the cervical nerves and carotid sinus reflexes. The effects of asphyxia-related circulatory reactions in blood chemistry can be objectified by demonstrating a difference between cardiac and sinus phosphatides (41/158). Likewise, the increased free histamine content of the hanging furrow represents a vital reaction (56/250). Such biochemical findings are diagnostically significant because bleeding in the soft tissues of the neck also occurs as congestion haemorrhage where there was no local force (64/147).

The articles on *death in water* are dominated by studies on the detection of diatoms used to diagnose death by drowning. Through drowning experiments in a diatomaceous earth suspension, Berthold Mueller and Dietrich Gorgs (39/715) were able to demonstrate that diatoms pass into the heart during the drowning process and, in addition, can also be detected in the systemic circulation, mainly in the liver. Further studies were followed, and by the end of the period under review, a total of 17 publications on the diatom problem were available. They presented sophisticated examination techniques for the detection of diatoms in the body, for example by phase contrast microscopy (46/235), but also discussed the evidential value of both positive and negative findings on the corpse. It was experimentally possible to prove that diatoms can penetrate post-mortem into the periphery of the lungs, which was contrary to the view prevailing until then (41/400). Finally, diatoms have also been found in corpses that had not been lying in the water (54/267, 64/21). Of the few other articles on death in water, the publication *New investigation method for drowning death* (63/134) by Herbert Reh is worth mentioning. His microscopically developed analysis of the lattice fibre texture of the alveolar septa allows conclusions to be drawn about a pre-existing emphysema aquosum. Such an examination allows the diagnosis of a typical drowning death even in severely decomposed bodies recovered from water. With his publication *Über das Blaukommen der Taucher (Morbus caerulescens)* [barotrauma of descent—lung squeeze] (39/378), Hermann Roer introduced this term into the forensic literature and at the same time triggered a controversy about the pathophysiology (40/252, 40/261).

Only a few publications have appeared on the major issues of investigation in *gunshot wounds*. A paper on the characteristics of the bullet entrance wound contains a collective case study of proven long-distance shots in which typical radial bursting of the skin was observed, as in a contact shot (61/48). As the mechanism of origin, it is assumed that bone splinters at the bullet entrance site are flung back from the inside to the outside. A case report on a homicide also concerns a bullet entrance wound that appeared to be the result of a contact shot (66/153). However, through numerous test shots, it could be shown that the unusual bullet entrance wound had been caused by a deformed bullet in a ricochet shot. Experimental studies on the effects of handguns have shown that, in addition to the direct impact and centrifugal effect, pressure differences can be demonstrated which have a suction effect in the direction opposite to the movement of the projectile, even when no contact shot is fired (45/414). As a result, tissue particles, debris and foreign bodies are sucked into the bullet track from the area of the exit wound (47/603). To determine the nature and extent of the backspatter of blood and tissue particles onto the shooter’s clothing or body, test shots were fired with a pistol (54/258). From the experiments, evidence as to the direction of the shot could only be derived for contact shots, while the images for near close-range shots were inconsistent. Several publications on gunshot injuries deal with the effects of small calibre weapons and captive bolt guns. In addition, a study was published on the occurrence of signs of close-range shots when using practice ammunition (56/1). As was to be expected in the post-war period, late effects of war injuries were reported in 3 publications. The majority of these are bullet and splinter injuries (52/399, 58/231), while the paper on ‘deaths from air raids’ (42/282) naturally also describes other consequences of violence in addition to such injuries.

For the assessment of *electrical fatalities*, the physical laws of electrical energy have to be considered in conjunction with the physiological and pathological-anatomical consequences for the human organism. This complex of problems was comprehensively presented in a critical review under the title ‘The medico-legal diagnosis of electrocution’ (47/29). The most important finding is the electric mark, the detection methods of which have been the subject of 8 publications. In addition to macroscopic examinations and examinations using a magnifier microscope (56/318), analyses with polarized light (57/431) as well as histological and histochemical techniques (56/269, 58/166, 62/26) were used to reliably identify a pathological skin change as an electric mark. Differences of differential diagnostic significance between electric and thermal marks have been thoroughly investigated by Ekkehardt Böhm in several experimental studies. The criteria are specific morphological characteristics (59/22, 61/128) and additionally metallisations of the skin (59/26, 63/149). As the case report on an electrical injury of the oral cavity (39/349) shows, changes resembling an electric mark also occur on the mucous membranes. The physiologist Hans Schaefer has described in detail the manifold consequences on the internal organs when an electric current flows through a body (47/5). Death from high-voltage current can occur as a result of exposure to technical electricity with corresponding voltage values (56/81) and also from natural causes in the event of a lightning strike (40/298, 44/743).

The physical damage caused by *heat* manifests itself in typical externally visible changes, but also on the internal organs. In a study on ‘corpses shrunken by heat’ (43/428), experiments have shown that the heat-induced shortening of the tendons and not of the muscles is the decisive factor in the post-mortem shrinkage processes on the corpse. Most of the publications reviewed concern thermally induced changes in the lungs. In animal experiments, serous inflammatory changes of the lung parenchyma and severe signs of decay of the elastic fibres could be observed as vital reactions after hot air inhalation of 600 ºC (45/394). In 3 papers on changes of the lungs after exposure to fire (49/130, 49/147, 49/382), the question was to what extent fat and soot particles in the lungs are to be regarded as an indicator of vital burning. The ‘brain damage in an acute fire-related death’ and its consequences for the dying process are extensively explained in a paper (41/129).

Only one paper is available on organ changes due to *cold*. It describes experimentally produced pulmonary haemorrhages after local exposure of the open brain of the experimental animals to cold (45/236).

In a case report on death by *starvation*, the killing of infants by deprivation of food is reported (64/93).

In 1958, a series of studies on human bones conducted in Kiel on health damage caused by *radiation* showed a level of strontium 90 in young people that was higher by a factor of about 10 compared with the age groups from 20 to 90 years (49/242). The detected long-lived β-emitter originated from the fallout of atomic bomb experiments, the health risks of which were still largely unexplored at that time, especially with regard to the genesis of malignant diseases. Table 2 gives an overview of the number of publications on all types of violent death.

**Table 2:** Articles on violent death published from 1948 to 1969

Violent death	Number of publications
Blunt force	31
Sharp force	13
Asphyxiation	28
Death in water	26
Gunshots	21
Electricity	20
Heat	7
Cold	1
Starvation	1
Radiation	1
Total	149

The speciality of *forensic obstetrics* covers the medico-legal problems of pregnancy and birth as well as the physical condition of new-borns. There are 4 papers on the course of pregnancy, one of which concerns the influence of electrical accidents and of lightning strikes on the development of gravidity (56/101). Seven papers deal with the determination of body length and age of human foetuses on the basis of different skeletal parts. With the exception of the first published article (40/567), all publications are by the Hungarian authors István Gyula Fazekas and Ferenc Kósa. In systematic studies, they analysed data from some flat bones (58/127), limb bones (58/142), skull base (60/48), skullcap (60/149), facial bones (61/13) and some small bones (61/29). Seven publications deal with birth trauma and postnatal injuries of new-borns, the main focus of which is the differential diagnosis of infanticide and spontaneous physical damage. Six publications have appeared on questions arising from Sect. 90 of the Code of Criminal Procedure on the opening of the corpse of a new-born child. Practically usable proof of life birth can be found in the umbilical region (61/117, 65/1, 66/35). The achieved status of the histological lung test around the mid-1950s is presented in a review (45/386), which was preceded by systematic studies on the development of elastic fibres in the human lung (45/381). The authors point out that microscopic examination of the lungs, in particular the morphology of the elastic fibres, can be of decisive importance in determining infanticide. Finally, a study is available on the gas content in the lungs and the gastrointestinal tract of new-borns of different levels of maturity (65/73). Radiological and autopsy examinations showed that as a result of artificial respiration with a mask, considerable amounts of air can enter the lungs, the stomach and also the intestines. There are 12 publications on practices, health hazards and proof of *abortion*, the majority of which date from the post-war period. In a compilation of cases on filicide by the mother (51/1), homicides of children aged between 6 weeks and 23 months are described.

In a review of *medical malpractice* (42/349), the medical issues and tasks of the medical expert are primarily explained. Of the individual topics, the 7 publications on the dangers of blood transfusions form the largest group. Of these, 3 papers deal with the classical problem of transfusion incidents due to incompatibility of group characteristics (40/363, 42/50, 43/38); another 3 papers address various health problems caused by transfusion (40/606, 43/562, 46/362), and one case report concerns transfusion syphilis (43/263). Vaccinations leading to death in children are the subject of 2 publications (41/405, 56/66), and 2 papers also deal with fatal complications of tracheotomy (58/40, 64/39). Under the title ‘Mors in tabula’ (42/385), Wilhelm Holczabek described such deaths from the autopsy material of the Vienna Institute in the first post-war years. A case report describes a death due to bilateral pneumothorax during plexus anaesthesia (42/438). The heterogeneous group of cases involving damage caused by toxic substances includes complications following the administration of drugs and contrast media. Except for insulin (44/386), these preparations are no longer in use today.

By far the most frequently described diseases and *sudden deaths from an internal cause* in adults were due to pathological changes in the cardiovascular system. Of these, 10 articles deal with findings in coronary heart disease, 3 with congenital heart and vascular malformations and 2 with myocarditis as the cause of death. In all deaths in a collective case study, ‘sudden cardiac failure was undoubtedly caused by the changes in the conduction system of the heart’ (63/199). Among the 8 publications on brain diseases are 2 articles on the forensic significance of epilepsy (46/212, 64/173). Four publications report on rather rare diseases of the gastrointestinal tract. From the broad spectrum of other pathologies, the sudden deaths from influenza in the 1950s are worth mentioning (43/44, 47/291). Compared to the adult age, only very few reports on sudden natural deaths of children appeared, with a total of 8 publications. Among these, only the diagnosis of inflammatory changes of the respiratory tract was found several times, namely in 3 cases. In 4 deceased children, various rare diseases were the cause of death. In another article, bacteriological findings were presented (56/167), which may contribute to the clarification of the pathologies in sudden deaths of children.

### Forensic toxicology

Under the title *Problems of Forensic Toxicology* (56/125), Emil Weinig presented a comprehensive overview in which the situation in the mid-1960s is critically examined. The first part deals with the significance of this special field within forensic medicine; the second part discusses toxicological analysis, taking into account all the proven special methods after sampling, separation, identification and evaluation; the third part addresses in detail the influences of biological and biochemical processes on the interpretation of the measurement results; and the fourth part calls for systematic scientific research in close cooperation between the forensic pathologist and the chemist with expertise in toxicological analysis.

There have been 37 publications on the *clinical signs of poisoning*. In addition to toxicokinetics, the majority of them describe primarily the symptoms of poisoning. The small group of 11 publications on inorganic poisons includes a first report on ‘6 years of experience with detoxified cooking gas’ (58/122). Among the 26 papers on organic poisons, 11 articles are about plant poisons, of which nicotine is the most frequently mentioned. In addition, a study on experimental nicotine intoxication (49/169), 2 compilations of cases from abroad (54/171, 54/367) and a case collection on ‘Three murders with nicotine’ were published (55/40). On poisoning with insecticides such as parathion (E 605®), there was a review ‘on the toxicology of some new pesticides’ (40/335) and 2 animal experimental studies on gastric peristalsis in parathion intoxication (56/148, 60/28).

Irrespective of the necessary toxicological and chemical analyses, the determination of *pathological anatomical findings* in tissues and organs is part of the diagnosis of poisoning. In 28 papers, morphological findings in cases of poisoning with agents from different substance groups are presented. Several publications concern carbon monoxide causing changes in the spleen (40/392), the brain (43/408, 56/74), the liver (52/258) and the heart muscle (52/357, 52/549). In acute fatal nicotine poisoning, morphological changes in the kidneys, the brain and the lungs can be found (54/304). The damaging effect of nicotine on the organism is also shown by changes in the blood count (56/62) and the eyeground (61/95). The morphological findings in parathion poisoning were studied in salivary glands (45/510) and in human oocytes (58/248). In cases of poisoning with the death cap (Amanita phalloides), typical histological changes of the liver are seen (50/417), which is a valuable additional finding in diagnosing the phalloidin syndrome.

The broad spectrum of potentially toxic substances requires a differentiated arsenal of methods for the detection of poisoning by *toxicological chemical analyses*. Accordingly, the 61 publications on poison analysis describe procedures for various substances. From a methodological point of view, there are papers on the ‘*Testfleckenmethode*’ [test spot method] (41/243), the crystal precipitation method (43/252), the chromometric gas analysis (45/68, 51/627), gas chromatography (52/22, 58/55), thin layer chromatography (52/605, 60/1), spectrophotometry (54/320) and the flame detector (63/154). In terms of the material to be investigated, the techniques for the detection of carbon monoxide dominate with 11 papers. Three articles have been published on the analysis of poisoning with phosphoric acid esters such as parathion (42/449, 47/580, 49/253). Noteworthy are 2 individual publications from the end of the 1960s on the problem of doping with metamphetamine (64/235) and on the use of the hypnotic drug methyprylon for knockout drops (65/44). Furthermore, it is noticeable that in addition to narcotics analysis (41/246, 41/420), the detection of drugs of different groups of active substances is addressed relatively frequently in 15 publications.

Of the articles on *alcohology*, a first thematic focus of 49 publications is on alcohol metabolism. In these studies, problems of all phases of the pharmacokinetics of alcohol are examined. Some special circumstances in the absorption phase are pointed out in 3 papers (40/399, 41/474, 45/401). Regarding distribution, there are studies on the distribution of alcohol in the blood (46/10), on the importance of body water for the distribution in the organism (46/53, 50/429) and on the distribution of alcohol between water and body fat (49/235). Questions of elimination are dealt with in 3 papers, one of which concerns the enzymology of alcohol metabolism (42/555) and the other two the varying values of alcohol breakdown (49/388, 50/228). The factor β_60_, which is significant for the expert opinion, varies depending on the physical condition of a person. Apart from malnutrition in the post-war period (39/84, 40/224), β_60_ values deviating from the average are observed after the impact of external force (43/18, 47/599) and depending on the blood alcohol concentration (44/374). The differently influenced metabolic processes inevitably affect the course of the blood alcohol curve, as shown in 6 papers. These include skull injuries (44/610), blood loss (44/615), shifts of blood and body water (46/744, 47/276), ‘accident-related stress effects’ (48/4) and drinking habits (55/265). Among the 4 publications on the toxic effect of methanol is a paper on the metabolic processes involved in the development of poisoning based on pathological anatomical findings.

The second thematic focus includes 52 publications on alcohol analysis of blood samples, body fluids and breath. Among them are articles on all routine methods of forensic alcohology. Various modifications have been described for the Widmark method (40/318, 41/164, 44/771). Several publications are also available on the ADH method (41/15, 48/26, 53/28). In the period under review, one publication on blood alcohol determination using only the gas chromatograph appeared (62/212). With regard to the sources of error of the individual methods, the analytical results of the Widmark and ADH methods were compared using different sample materials (48/393, 48/400, 48/403). In another comparative study, gas chromatography was included as a third method (54/150). As with the analytical methods, there have also been systematic studies on the validity of alcohol determination in various blood samples. Experiments with putrid blood have shown that both alcohol loss and new alcohol formation can occur (41/424, 42/75, 43/221). Studies on blood samples stored for longer periods (47/614, 50/34, 52/76) and on sera stored for longer periods (52/424) showed a decrease in alcohol content with increasing storage time. Blood alcohol determination on coagulated blood yielded lower alcohol concentrations compared to whole blood and blood serum (49/113, 49/431). For correct determination of the blood alcohol content in the corpse, blood must be taken from a femoral vein because of the post-mortem diffusion of alcohol in the abdominal cavity (46/357, 46/735, 47/619). A large number of papers deal with the determination of the blood alcohol concentration in the presence of additional exogenous noxious substances, e.g. by inhalation of propellant gas (39/280) or by oral ingestion of camphor (47/289). A larger study led to fundamental findings in the evaluation of alcohol results in the cerebrospinal fluid (48/257). Apart from the fact that alcohol is neither normally detectable in cerebrospinal fluid nor formed post-mortem, the cerebrospinal fluid/blood alcohol concentration is forensically significant for the assessment of the metabolic phase of alcohol. In a report, first practical experiences with the breath alcohol tube Alcotest® are described, which were collected on several hundred test persons (48/417). The test tube provided reliable results when used as a pre-test.

### Identification of unknown decedents

The majority of publications on *identification methods* concern forensic osteological findings. Two microscopic comparative anatomical studies are available to distinguish human and animal bones (43/273, 44/578). There are several regions on the human skeleton that allow sex estimation (47/442, 54/227), age estimation (41/147, 51/161, 58/205) and reconstruction of body height (42/189, 48/378). In a complex investigation to determine the resting time of skeletal remains, a wide range of macro- and micromorphological, chemical and physical methods were used (47/209). Among the other papers are several case reports and some studies on the suitability of various characteristics for the identification of unknown dead bodies, such as the microscopic detection of sex chromatin (46/242) and a photographic method for skull identification (48/247).

### Forensic genetics

Table 3 provides an overview of the number of publications on forensic genetics.

**Table 3:** Articles on forensic genetics published from 1948 to 1969

Topic	Number of publications
Blood typing	
Test methods	31
Occurrence and distribution	10
Inheritance	14
Trace analysis	
Blood detection	3
Species specificity determination	9
Individualisation	18
Sperm traces	6
Hair traces	7
Other	6
Parentage testing	
Procreative capacity	3
Hereditary biology	10
Blood groups	28
Total	145

A comprehensive review by Franz Josef Holzer (42/416) shows the rapid progress of *blood group science* in the half century since the discovery of the AB0 groups in 1901. This increase in knowledge is reflected in the publications on test methods, occurrence and distribution as well as inheritance of the erythrocytic systems and factors. Of these, 10 studies deal with the AB0 groups, 8 with the A subgroups, 8 with the Rh system and 5 with the MN system. In the results of an experimental study, it is reported that the antigens A, B and M, N can be reliably detected using the mixed cell agglutination method on bone, muscle and kidney tissue (63/162). As a ‘new agglutinable blood cell trait’, the S trait was added (40/160), which represents an extension of the MN system. The ‘Q-blood factor’ (44/356) is interpreted by the authors as an independent trait, but according to the majority opinion, it is identical with the P-factor (46/46, 55/103). Another paper contains methodological guidance on ‘securing Kell findings’ (19/59), which are rare blood group characteristics.

The haptoglobin types used in early forensics belong to the human *serum groups*. In the 4 papers on this system, different electrophoretic techniques are described for the depiction of the Hp groups. Three publications each appeared on the immunoglobulin system Gm, a gamma chain marker and on the Gc system, a group-specific component found in the α_2_-globulin region. Lipoproteins also show a genetically determined polymorphism (60/90), but the Lp system proved to be problematic in practical application.

Of the papers on *erythrocytic enzyme groups*, 5 relate to the polymorphism of erythrocyte acid phosphatase. The two articles first published on the enzyme system abbreviated acP were by Georg Radam and Hansjürg Strauch (60/39) as well as by Otto Prokop (61/59) from the Institute of Forensic Medicine in East Berlin. The only paper on the genetically controlled isoenzyme patterns of adenylate kinase (63/166) was also published by Radam and Strauch, in which they presented a method for determining AK types that is suitable for forensic purposes.

In *trace analysis*, the examination of material with suspected traces of blood still required the hierarchical sequence of steps according to the scheme: attempt to detect blood — determine species specificity — identify group characteristics. The 3 articles on the *detection of blood* are concerned with the acetone-chlorohaemin reaction (39/495) from the group of crystal reactions and the porphyrin test (44/550, 45/370). Among the publications on the *determination of species specificity,* descriptions of the precipitation reaction according to Uhlenhuth, whose personal memoirs were published at the beginning of 1949, dominate (39/309). An antiglobulin test (45/527) and a micromethod for the detection of eserine-sensitive esterases (61/137) were developed as alternative techniques for the detection of human blood. Other papers are devoted to special research questions, such as the determination of the age of blood traces (40/478) and the detection of birth blood (45/62). In the publications on the *individualisation of blood traces*, hereditary characteristics of agglutinable erythrocyte systems as well as serum and enzyme groups are described. The status reached at the end of the period under review is summarised in a critical analysis of blood groups used in trace analysis (66/130). The papers on agglutinable traits show that in addition to the AB0 and MN systems, the factor P (41/451) and the A subgroups (45/355) can also be used in forensic trace investigations. Of the serum groups, the Gm system (53/18) has proved to be particularly suitable for blood trace investigations. Among the erythrocytic enzyme groups, the phosphoglucomutase (PGM) types (66/31) are among the preferred and proven isoenzyme patterns.

For the examination of *sperm traces*, in addition to the crystal reactions, which are susceptible to interference, there is the detection of acid phosphatase as an enzymatic laboratory test sample (49/5, 53/175, 66/1). Another publication (42/605) describes methods for the detection of traces of semen, vaginal secretions and blood of genital origin. The only article found on group traits contains the results of a series of experiments on the typing of the Gm^a^ trait in human semen (52/610).

The examination of *hair traces* often involves the demonstration of external influences on the hair, for example heat (39/1) or cosmetics (40/553). One article has been published on the occurrence of the group substances A, B, 0 and AB in human head hair (42/395). Steffen Berg has critically discussed the range of methods and the interpretation of findings in forensic hair examination in a detailed overview (46/531).

Only a few publications deal with meconium traces (56/57), saliva traces (56/221) and urine traces (58/222).

In the period under review, *parentage testing* comprised the forensic assessment of fertility, but also morphological and functional hereditary traits and, increasingly, genetically determined group characteristics of erythrocytes and blood serum. Among the publications on usable blood group systems, the 16 papers on agglutinable erythrocyte characteristics form the largest group of topics. While mostly individual blood group systems and their evidential value are considered, an article by Paul Speiser illustrates the status around the mid-1950s (45/50). Using the criteria A_1_, A_2_, B, 0, A_1_B, A_2_B, M–N, S, P, K, F_y_, C–c, D-dd, E-e, Lewis and secretor status, a general paternity exclusion probability of 70% was achievable. This value represents a significant achievement, as when A_1_, A_2_, B, 0 and M–N were used alone; the general paternity exclusion probability was 30% just a few years earlier. Since the 1960s, the serum groups Gm (53/122, 58/261), Hp (53/186) and Gc (62/261) as well as the acP polymorphism (64/127) have been typed in cases of disputed paternity. The mathematical and statistical problems of parentage testing are the subject of 7 articles. Two of these papers compare the calculated probability based on the blood group characteristics with the genetic expert opinion. While the older publication of 1958 (47/484) holds the view that the Essen-Möller values ‘never permit a valid conclusion in isolation’, the more recent work of 1968 (63/53) argues in favour of ‘the routine application of the Essen-Möller procedure’.

### Scientific-technical criminalistics

In the years since the end of the war, only 11 publications from 3 subfields of scientific-technical criminalistics were found. There are 6 articles on *ballistics*, which, in addition to problems of trace analysis, deal with methods of determining the firing distance by spectrographic analysis (56/39) and by radiographic examination (65/112). The four papers on *forensic biology* concern the use of luminol in forensic practice (57/410, 64/158) and traces of semen on textiles (44/781, 60/42). The subject of the ‘*Signalementslehre*’ is the methodology of describing persons, the characteristics of which also include dynamic features such as gait (43/11).

### Clinical legal medicine

Only a few articles deal with forensic findings on living persons. Two publications describe survived injuries caused by *external force*. One is about a war injury with subsequent bullet embolism (39/139), and the other is about the burning of a woman’s genitals by her jealous husband (66/13). Causes and consequences of *drug addiction* are reported in 2 articles where cliradone (43/242) and codeine (47/246) were the abused substances. A collective case study (64/229) contains several cases of *self-harm* by means of artificially induced subcutaneous skin emphysema of the face, which was intended to simulate trauma or illness.

### Forensic psychiatry and psychology

Wilhelm Hallermann considered a fundamental problem of *psychopathology* in his essay *On the change of the concept of psychopathy* (51/588). In this context, the multifaceted ‘psychopathology of the arsonist’ is a special case (49/64). Other aetiological factors discussed in several articles with regard to becoming a criminal are delays in psychosexual maturation and psychiatric illnesses. Two papers on the significance of depth psychology and narcoanalysis have been published on the *psychology of evidence* (41/360, 41/375). The use of psychotropic drugs in criminal proceedings is rejected by both lawyers and psychiatrists as a prohibited interrogation method because of its ‘inadequacy and ethical reprehensibility’.

Most of the publications on *suicidology* are concerned with various factors influencing the decision to commit suicide, such as ‘foehn and suicide’ (47/271), ‘alcoholic motivation’ in suicides (55/67) and ‘suicide and pregnancy’ (56/172). The transcript of the ‘tape recording of a suicide’ by inhalation of coal gas after previous ingestion of sleeping pills (58/18) was published by Gerhard Dietz. The compilation of cases ‘Suicidal jumps from the Little Belt Bridge’ (63/223) is another example of the preference for certain building structures to commit suicide.

### Sexual medicine

The publications on *paraphilias* are primarily concerned with incest relevant to criminal law, whereby this delinquency of the post-war period is analysed as a peculiarity of the time (42/452, 44/259). One case report deals with the repeated sexual abuse of ‘1 to 5-month-old infants by a paedophile’ (60/163), who was assessed by an expert as a psychosexually infantile offender. Another crime is described in the report on the rape of a 10-year-old girl by a 15-year-old youth (54/231). In the 2 publications on *auto-erotic accidents*, similar deaths by electric current during sexual activity are reported.

### Traffic medicine

Under the title *The present status of blood alcohol research* (41/1), Berthold Mueller published a review paper on the problems of *alcohol and traffic safety* in 1952. In addition, 14 papers deal with individual issues of alcohol-related impaired performance in drivers and pedestrians. Other influences on road safety are described as feeling tired or falling asleep at the wheel (44/343, 45/523), spontaneous (44/594) and drug-induced hypoglycaemia (64/217) and short-acting narcotics (49/187). Another study explains how the ‘change in performance in old age’ (49/100) affects the fitness to drive. According to the results of a Munich study (49/187), sudden cardiac death in connection with road traffic became increasingly important as early as the 1950s. The sequels of road traffic injuries are the subject of 11 publications explaining patterns of injury to the head, neck, chest and legs. Other publications include reviews of the necessary examination findings and investigation results to determine the position of the occupants and in particular the driver in the vehicle at the time of the accident (49/247) and to reconstruct the course of events (63/218).

### Social medicine

In 3 of the only 5 publications, the focus is on *occupational activities* and specifically on occupational diseases of workers in corundum plants (39/577) as well as of miners and other workers exposed to the risk of silicosis (49/194, 54/379).

### Criminology

Of the subfields of criminology, *criminal aetiology* is represented by 2 publications. One article is entitled *The film as a template for capital crimes* (48/576), and the other paper analyses the ‘criminogenic significance of loneliness and isolation’ (51/595). The only publication on *criminal phenomenology* concerns ‘criminality in the brain-injured’ (43/59). Likewise, only one article (53/55) has been published on *victimology*, with which the authors have made a contribution to the analysis of the victim types of persons who have been killed. The criminological analyses of individual *offence groups* are devoted to the special features of juvenile delinquency (46/555, 48/17, 51/18) and the fight against drug addiction (44/14). Publications on *prisons and the penal system* include 2 compilations of cases of rare self-harm in prisons (39/529, 43/545) and a report on criminal biology investigations in the Baden juvenile penal system (42/1).

## Discussion

The period under review begins a good 4 years after the end of the Second World War and extends over a little more than 2 decades until the turn of the year 1969/1970. The aftermath of the war led to sometimes serious social problems, which inevitably had an impact on crime and consequently provided the motivation for scientific studies on contemporary issues (42/452, 44/259). On the other hand, some publications illustrate the difficulties of fighting crime under the conditions of the post-war period (41/267, 41/345). Overall, the publications that appeared in the analysed period show a change in the professional profile of forensic medicine, which is characterised by a departure from both the psychological and psychiatric content and the scientific-criminalistic orientation of the discipline, with a simultaneous increased focus on morphological and even more on haemogenetic topics.

The history of forensic medicine in Germany during the twentieth century is characterised by changing development trends, which have led to links with social medicine, with insurance medicine and not least with scientific and technical criminalistics. Logically, Wolfgang Schwerd argued in the explanatory memorandum to the change of the name of forensic medicine to legal medicine: ‘This name change took place in the context of comprehensive efforts to rethink the position and tasks of the discipline and — where necessary — to reform it. These considerations initially focussed on efforts to reformulate the discipline’s spectrum of tasks in teaching, research and practice in order to provide the Federal Ministry of Health, the Science Council and the West German Medical Faculty Association with a clear idea of the discipline’s concerns for their work on the reform of the study of medicine and on the new licensing regulations’ (61/65). This well-formulated intention is nothing other than part of the on-going struggle of legal medicine for its existence, since the Science Council, in its ‘Recommendations for the Reorganisation of Studies at Universities’ of 14 May 1966, had defined the concentration of the curriculum as an essential goal of a reorganisation of clinical studies and arrived at the following conclusions: ‘It is therefore recommended that teaching in the main clinical subjects be intensified, while that in the other subjects be streamlined or, as far as possible, incorporated as an integral part of teaching in the basic clinical subjects. According to this, subjects such as […] legal medicine […] should no longer be taught as special subjects in special lectures and exercises, but as part of the complex of internal medicine, surgery or other integrating subjects of clinical medicine’ [6]. The implementation of this recommendation would have meant a return to the nineteenth century for the discipline, because it was only after many years of efforts by renowned representatives of the field that forensic medicine was included as a compulsory subject in the study of medicine in 1901 [7].

In the post-war period, it was necessary to eliminate the National Socialist injustice through changes in the law and thus to restore the basis of the rule of law for the fulfilment of the forensic and medical tasks. An example of this is the Marriage Act, enacted as Control Council Act No. 16 on 20 February 1946, from which the terms mental disorder and mental illness are explained in a publication (45/420) in accordance with Sects. 44/45 of the Marriage Act. In spite of the decades-old demand for the introduction of officially ordered autopsies in Germany [1], in contrast to other European states (63/171), there was still no uniform legal regulation on the extrajudicial post-mortem examination in the Federal Republic.

For some injuries, the routine technique of opening a corpse is not sufficient, but a special dissection technique is needed. With the increasing motorised road traffic, fatal accidents involving whiplash injuries of the cervical spine were on the rise. The subtle examination method described (64/204) makes it possible to record all pathomorphological findings on the soft tissues of the neck, the spinal column and the spinal cord. Although the mandatory introduction of head restraints since the end of the 1960s has succeeded in significantly reducing serious injuries to the cervical spine in accidents, vehicle occupants still suffer such injuries in collisions.

In his critical review ‘on rigor mortis’ (39/186), Wolfgang Laves raised conclusive objections against the lactic acid hypothesis and, on the other hand, used the biochemical processes in nucleotide metabolism to explain the development of post-mortem rigidity. This ‘shattered the foundations of the lactic acid theory of rigor mortis’, so that this explanation is now only of historical interest. Experimental investigations have demonstrated the crucial importance of the redox potential for the decomposition processes in putrefaction and decay (41/236). Accordingly, both decomposition processes ‘essentially differ only in the level of their redox situation’. The increasing use of sulphonamides and antibiotics in clinical medicine has led to the question in thanatochemistry whether the administration of such preparations has an inhibitory effect on bacterial decomposition processes. The results of animal experiments with tetracycline (51/572) suggested that the administration of antibiotics both reduces the bacterial flora involved in the decay processes and inhibits proteolytic enzymes. Consequently, such exogenous influences must be taken into account in a time-related assessment of the degree of decomposition of a corpse. The varying levels of adrenaline metabolites and histamine in corpse blood seen in different causes of death represent a vital reaction (54/136). In spite of the short intravital half-life of catecholamines, they can be detected in corpse blood for an astonishingly long time and allow to differentiate between violent death mechanisms with short agony and reflex deaths without corresponding processes.

Several publications on the effects of blunt force describe some fracture mechanisms that are of importance for expert opinions. At that time, it was widely believed about the ring fracture of the skull base that such a circular fracture of the posterior cranial fossa occurs when the spine is forced into the base of the skull as a result of an impact. In addition to this, one publication (51/601) refers to the previously largely ignored fracture formation due to the effect of traction forces between the base of the skull and the spine. Moreover, it could be shown in a model test (52/13) that an annular extension fracture of the skull base occurs in a vehicle impact from behind at high speed and in the presence of a thin skull.

New findings have also been published for the assessment of the sequelae of sharp force. These include the wound morphological characteristics of injuries caused by scissors (45/53). Through numerous stabbing experiments, Joachim Rauschke found out that typical tissue tongues develop when the wound is caused by closed scissors, which protrude into the lumen of the track of the stab wound next to the wound angles. The stab wounds caused by a single scissor blade show different histological features than stab wounds due to a knife. As model tests by Kyrill Bosch with different knives (54/273) showed, the wounds allow specific statements about the shape of the blade, i.e. saw structures can be distinguished from serrations. He supplemented these basic research results with another extensive experimental study using various piercing tools (62/4).

For confirming the cause of death by hanging, various biochemical findings are suitable to prove a vital course of events. According to a study by Steffen Berg (41/158), the manifestation of cardiovascular reactions to asphyxia in blood chemistry can be demonstrated by a difference between cardiac and sinus phosphatides. The significantly greater free histamine content in the hanging furrow compared to the free histamine in the intact neck skin of the same corpse is diagnostically useful as a vital reaction, as István Gyula Fazekas and Elisabet Virágos-Kis (56/250) have found. Such biochemical detection methods can help to objectify neck compression, especially since bleeding in the soft tissues of the neck also occurs as a result of congestion without local force (64/147).

As a result of numerous studies, the opinion on the evidence value of diatoms in the diagnosis of drowning has gradually changed. At the beginning of the many years of work on this problem was an animal experiment by Berthold Mueller and Dietrich Gorgs (39/715). Through drowning experiments in a diatomaceous earth suspension, they were able to prove ‘that diatoms up to a size of about 30 µ pass into the heart during the drowning process (though in relatively small numbers), and that they can also be found in the systemic circulation, mainly in the liver, and occasionally also in the brain and kidneys’. Further experiments (41/400) showed ‘that, contrary to the hitherto prevailing view, fluid components (diatoms) can penetrate into the periphery of the lungs post-mortem, especially when the corpses are moved in the water or when a higher water pressure rests on them’. After diatoms had also been found in organs of corpses that had not been in the water (54/267), Berthold Mueller wrote in summary: ‘On the whole, it seems that a refinement of the technique of detecting diatoms tends to make the diagnosis of death by drowning even more uncertain; […] It will be necessary to explore in detail the limits of the usability of the findings, taking into account local conditions.’ Subsequent investigations (64/21) confirmed the presence of diatoms in the systemic circulation of non-drowned persons, but in lower numbers than in drowned persons. As a way out of the diagnostic dilemma, the determination of a ‘minimum number of diatoms; was demanded, which, if exceeded, would be ‘usable as an essential clue for a drowning death’.

The term ‘Blaukommen der Taucher’ [barotrauma of descent — lung squeeze] (39/378), introduced by Hermann Roer, is still used today, and its explanation has also been confirmed in its basic features, despite exceptionally extensive criticism (40/252, 40/261). Massive facial cyanosis occurs mainly due to compression of the diving suit when the diver descends too quickly to a greater depth, causing a strong negative pressure in the helmet (‘suction in the helmet/lung space’).

In electrical fatalities, an electric mark may appear on the skin or mucous membrane as an indicative finding. Subtle diagnostic procedures are necessary to distinguish electrothermal damage from other thermal damage. In this context, a comprehensive spectrum of methods has been described, including macroscopic examinations and examinations using a magnifier microscope (56/318), analyses with polarized light (57/431) as well as histological and histochemical techniques (56/269, 58/166, 62/26). In several experimental studies, Ekkehardt Böhm investigated differences between electric and thermal marks that were significant for differential diagnosis. Morphological characteristics (59/22, 61/128) and additional specific differences in metallisation in the skin (59/26, 63/149) were found to be useful diagnostic criteria.

One of the striking changes in charred corpses is the heat-induced shrinkage of the body. In a series of basic experiments (43/428) on these changes, it was found that tendons shorten by a maximum of 61.5% of their original length on average under increasing dry heat. Consequently, ‘the tendons, not the muscles, play the decisive role in the processes that lead to the fixed dislocations of the extremities’. The criminal trial of the dentist Dr. Richard Müller for the fire-related death of his wife [8] was a cause célèbre not only for criminalistics, but also for forensic medicine. The medical experts had not been able to agree on the extent to which fat and soot particles in the lungs of burnt bodies are to be judged as being of vital origin. This controversy prompted several studies on lung changes after exposure to fire (49/130, 49/147, 49/382).

Beyond forensic obstetrical questions, the Hungarian authors István Gyula Fazekas and Ferenc Kósa also opened a new chapter for the identification of unknown dead bodies with their investigations on the determination of body length and age of human foetuses on the basis of various skeletal parts [9]. In systematic studies, data from several flat bones (58/127), limb bones (58/142), the skull base (60/48), the skullcap (60/149), facial bones (61/13) and some small bones (61/29) were analysed. A study on the gas content in the lungs and gastrointestinal tract of new-borns of different maturity showed the consequences of artificial respiration with a mask (65/73). Beyond the known experience that air in these organs is a sign of live birth, x-rays and autopsies showed that as a result of this method of ventilation, considerable amounts of air can enter the lungs, the stomach and also the intestines. Accordingly, such a resuscitation measure represents a source of error in the assessment of macroscopic autopsy findings.

In sudden deaths from an internal cause, there is a dominance of cardiovascular diseases among the publications reviewed, which has been unchanged in the cause of death statistics since the beginning of the 1950s [10]. The expected distribution can also be seen in this disease group, as coronary heart disease is by far the leading cause of death. In contrast to adult deaths, there are only a few publications on sudden deaths in childhood, which mostly relate to rather rare diseases. Systematic studies to elucidate the aetiology of sudden infant death syndrome could not be found.

Carbon monoxide intoxication was still one of the most frequent forms of poisoning during the period under review, and town gas, also known as cooking gas or coal gas, was usually the source of the poison. To counteract this situation, a detoxification plant was put into operation in Basel in 1958. The report on ‘6 years of experience with detoxified cooking gas’ (58/122) shows that no more suicides with cooking gas were recorded in the Basel Medico-Legal Institute during that period, but that fatal accidents continued to occur despite the gas conversion. Even with the conversion of the gas supply to natural gas, the risk of CO formation during incomplete combustion due to oxygen depletion in the atmospheric air remained, and there was also the risk that explosive natural gas-air mixtures might form [11]. Most publications on parathion (E 605®) appeared after the easily obtainable insecticide had quickly become a fashionable poison (‘Worms poison’) after 1954, when the series of murders of Christa Lehmann in Worms [12] became known and prompted copycat acts [13]. Publications on the symptoms of intoxication include 2 animal studies on gastric peristalsis in parathion poisoning (56/148, 60/28). The morphological changes caused by exposure to parathion were studied in salivary glands (45/510) and in human oocytes (58/248). Three articles were published on the toxicological and chemical analysis of poisoning with phosphoric acid esters such as parathion (42/449, 47/580, 49/253). Two individual publications from the end of the 1960s are noteworthy, as they contain early reports on doping with metamphetamine (64/235) and the use of the hypnotic methyprylon for knockout drops (65/44).

The studies on alcohol metabolism cover all phases of the pharmacokinetics of ethanol. The knowledge gained about special features of absorption (40/399, 41/474, 45/401), distribution (46/53, 49/235, 50/429) and elimination (49/388, 50/228) allowed the forensic assessment of alcoholic influence to be further elaborated. Several studies have shown that factor β_60_, which is significant for expert opinions, is not a constant, but varies depending on the physical conditions of a person. Deviating β_60_ values were found after the impact of external force (43/18, 47/599) and depending on the blood alcohol concentration (44/374). With rising blood alcohol content, the determined β_60_ values increased and reached a mean value of 0.203‰ at blood alcohol content above 2‰. For alcohol analysis, methods for determining concentrations in blood samples and body fluids with the routinely used examination techniques were described. These publications were aimed at minimising the sources of error of the original technique of the Widmark method [14], the ADH method [15] and gas chromatography [16] by methodological modifications. Direct gas chromatographic determination of the blood alcohol concentration (62/212) has proved to be a specific method with high reproducibility of the measurement results. The validity of the analytical results was also verified on different sample materials. Systematic studies on blood samples stored for longer periods (47/614, 50/34, 52/76) and on sera stored for longer periods (52/424) showed that the alcohol content decreases with increasing storage time. In contrast, both alcohol loss and new alcohol formation can occur with putrid blood (41/424, 42/75, 43/221). At the beginning of the 1950s, breath alcohol analysis was tested as a new examination principle, the development of which up to a technically reliable test procedure is the merit of Ernst Scheibe, who later became professor in Greifswald [17]. In a report (48/417), practical experiences with the breath alcohol tube Alcotest®, which has been produced since 1953, are presented, which were collected from several hundred test persons. From the laboratory test sample with the test tube, further development led to a conclusive breath alcohol measurement [18].

The spectrum of physical characteristics for the identification of unknown dead persons was enriched above all by osteological examination findings. The index acetabulo-ischiadicus (47/442) and the thickness of the skullcap are suitable for sex identification (54/227). In addition to pelvic bones (41/147) and femur (58/205), other skeletal parts and the teeth (51/161) can be used for age estimation. The estimation formulas for the reconstruction of body height were validated using the long limb bones (42/189) and the foot and hand length (48/378). Progress was also made in the examination techniques used to identify skulls. The original method of verifying the identity of skulls by graphic means [19] was followed by the photographic method (48/247), using an optical bench with a photo camera and a sighting frame. The unknown skull is aligned with the comparison photograph by means of anatomical auxiliary lines, and the photographic superposition of skull and comparison photograph is carried out by exposing the film twice.

Since the discovery of the AB0 groups by Karl Landsteiner in 1901 [20], a number of agglutinable characteristics of erythrocytes have been added to the classical blood groups. After the A-subgroups had been identified, the blood group characteristics M–N and P as well as the Rh system followed [21]. Through numerous studies, the knowledge about test methods, occurrence and distribution as well as inheritance of the erythrocytic systems and factors could be broadened and made usable for forensic purposes. The MN system was extended by a ‘new agglutinable blood cell property’, which was incorporated into the blood group nomenclature as feature S (40/160). The previously known ‘Q-blood factor’ (44/356) is regarded by the authors as an independent characteristic, but the majority considers it to be identical with the P-factor.

With the discovery of the haptoglobin polymorphism [22], the era of human serum groups began in 1955. Like the genetically determined Hp types, the immunoglobulin system Gm and the Gc system of α_2_-globulins have proved to be forensically useful.

Of the erythrocytic enzyme groups, the isoenzymes of erythrocyte acid phosphatase [23] were the first hereditary features to be discovered in 1963. Forensically important details about the enzyme system abbreviated acP were worked out by Georg Radam and Hansjürg Strauch (60/39) as well as by Otto Prokop (61/59) at the Institute for Forensic Medicine in East Berlin. Furthermore, in the only paper on the polymorphism of adenylate kinase (63/166), Radam and Strauch presented a method for the determination of AK types that is useful for forensic purposes.

In trace analysis, the acetone-chlorohemine reaction (39/495) from the group of crystal reactions and the porphyrin test (44/550, 45/370) were still in use for the detection of blood. The precipitation reaction according to Uhlenhuth was the preferred method for determining species specificity (39/309). A further individualisation of blood traces had become possible because, in addition to the AB0 and MN systems, factor P (41/451) and the A subgroups (45/355) could also be successfully typed on dried blood. Of the serum groups, the Gm system (53/18) and among the erythrocytic enzyme groups the PGM characteristics (66/31) had proved to be particularly suitable for tracing purposes. The group substances A, B, 0 and AB could be detected in human head hair (42/395) and the characteristic Gm^a^ in human sperm (52/610).

The rapid progress in haemogenetics was particularly evident in the assessment of parentage. In the 1920s, when the first expert opinions on blood groups in cases of disputed paternity were given solely on the basis of the AB0 system, the overall chance of paternity exclusion was 20% [24]. After the range of characteristics was gradually extended (45/50), the overall chance of paternity exclusion increased to 30% when the criteria A_1_, A_2_, B, 0 and M–N were used and reached a value of 70% in the mid-1950s with the blood group mosaic A_1_, A_2_, B, 0, A_1_B, A_2_B, M–N, S, P, K, F_y_, C–c, D-dd, E-e, Lewis and secretor status. A further increase in the overall chance of paternity exclusion has been achieved since the 1960s, when the serum groups Hp, Gm and Gc as well as the acP polymorphism were typed in cases of disputed paternity [24].

In the post-war period, research priorities in the Institutes of Forensic Medicine began to shift. As a result of this development, scientific work on problems relating to scientific-technical criminalistics took a back seat. Thus, the 113 out of 1762 publications on forensic topics in the period from 1922 to 1944 [1] contrasted with only 11 out of 912 publications from the period between 1948 and 1969. These are papers from the fields of ballistics, forensic biology and ‘Signalementslehre’, which address questions of both forensic and medical relevance.

The few publications available on forensic findings in living persons do not even rudimentarily reflect the diversity of topics in clinical legal medicine. They are all exclusive case histories describing individual cases of external violence, drug abuse and self-harm. Analytical papers on such widespread crimes like sexual violence and child abuse have not been published.

The shift in the research focus of the Institutes of Forensic Medicine since the post-war period has also caused a decline in the number of publications in the fields of forensic psychiatry and psychology. From 75 publications in the years 1922 to 1944 [1], the number dropped to 24 papers in the period reviewed here. The unanimous rejection of narcoanalysis as an accompanying measure during interrogations in criminal proceedings, as expressed in the two publications on the psychology of evidence, has persisted to this day.

The publications relating to sexual medicine complement the case reports on paraphilias, especially the phenomenology of the offences of incest and paedophilia. Similarly, the publications found on autoerotic accidents supplement the case reports on the appearance of electrocution during sexual activity.

The rapid increase in motorised road traffic created dangers that inevitably had an impact on the scientific problems in traffic medicine. A priority topic was research into alcohol-related performance impairments in drivers and pedestrians (41/1). In addition, endogenous and other exogenous influences on the fitness to drive were also investigated. The knowledge gained, for example on feeling tired and falling asleep at the wheel (44/343, 45/523) or on the influence of pharmaceuticals on road users (49/187, 64/217), was essential for the prevention of accidents. The analysis of sequelae of injuries after traffic accidents formed an essential basis for the forensic and medical assessment of accident victims. It often involved complex issues regarding the reconstruction of accidents (49/247, 63/218).

From the field of criminology, especially the articles on the aetiology of crime have a current relevance that can hardly be overlooked. The one article entitled *The film as a template for capital crimes* (48/576) brings to mind the intolerably accurate reports in the mass media that prompted concrete copycat acts [25]. In the other publication, the ‘criminogenic significance of loneliness and isolation’ (51/595) is analysed. At present, public debate often focuses on the manifold effects of the coronavirus pandemic, the consequences of which could go beyond disruption of mental balance in persons with certain personality traits.

## Footnotes

1 The first number refers to the volume, the second to the start page.
